# Targeting the DPP-4-GLP-1 pathway improves exercise tolerance in heart failure patients: a systematic review and meta-analysis

**DOI:** 10.1186/s12872-019-01275-5

**Published:** 2019-12-23

**Authors:** Chengcong Chen, Ying Huang, Yongmei Zeng, Xiyan Lu, Guoqing Dong

**Affiliations:** 1 Section of Endocrinology, Department of Pediatrics, Shenzhen Maternity & Child Healthcare Hospital, Shenzhen, China; 2 School of Public Health, Chinese University of Hong Kong, Hong Kong, China; 3 Section of Gastroenterology, Department of Pediatrics, Shenzhen Maternity&Child Healthcare Hospital, Shenzhen, China

**Keywords:** Heart failure, GLP-1 receptor agonist, DPP-4 inhibitor, Exercise tolerance

## Abstract

**Background:**

The most significant manifestation of heart failure is exercise intolerance**.** This systematic review and meta-analysis was performed to investigate whether dipeptidyl peptidase-4 (DPP-4) inhibitors or glucagon-like peptide 1 receptor agonists (GLP-1 RAs), widely used anti-diabetic drugs, could improve exercise tolerance in heart failure patients with or without type 2 diabetes mellitus.

**Methods:**

An electronic search of PubMed, EMBASE and the Cochrane Library was carried out through March 8th, 2019, for eligible trials. Only randomized controlled studies were included. The primary outcome was exercise tolerance [6-min walk test (6MWT) and peak O_2_ consumption], and the secondary outcomes included quality of life (QoL), adverse events (AEs) and all-cause death.

**Result:**

After the literature was screened by two reviewers independently, four trials (659 patients) conducted with heart failure patients with or without type 2 diabetes met the eligibility criteria. The results suggested that targeting the DPP-4-GLP-1 pathway can improve exercise tolerance in heart failure patients [MD 24.88 (95% CI 5.45, 44.31), *P* = 0.01] without decreasing QoL [SMD -0.51 (95% CI -1.13, 0.10), *P* = 0.10]; additionally, targeting the DPP-4-GLP-1 pathway did not show signs of increasing the incidence of serious AEs or mortality.

**Conclusion:**

Our results suggest that DPP-4 inhibitors or GLP-1 RAs improve exercise tolerance in heart failure patients. Although the use of these drugs for heart failure has not been approved by any organization, they may be a better choice for type 2 diabetes mellitus patients with heart failure. Furthermore, as this pathway contributes to the improvement of exercise tolerance, it may be worth further investigation in exercise-intolerant patients with other diseases.

## Background

Heart failure (HF) is a complex clinical syndrome resulting from structural or functional impairment of ventricular filling or the ejection of blood, and the lifetime risk of HF ranges from 20 to 46% [1]. Those with diabetes mellitus have at least double the risk. HF patients with diabetes are associated with a higher rate of hospitalization and mortality [2, 3]. Although mortality has declined due to the use of angiotensin-converting enzyme inhibitors, beta-blockers and spironolactone [4], approximately 50% of patients die 5 years after diagnosis [5]. Standard treatments for patients with HF and diabetes are lacking [6]. Moreover, HF results in another serious issue among patients. Dyspnoea and fatigue, the most common symptoms among HF patients, can result in exercise intolerance [5], which not only contributes to decreasing quality of life (QoL) among patients but also results in impairment of working capacity, further increasing the economic burden [7]. However, very few studies have focused on exercise tolerance in this population.

A body of literature demonstrates that lower health-related QoL is associated with a higher mortality rate among HF patients [5, 8]. In particular, QoL was significantly reduced in HF patients with exercise intolerance. Thus, improvements in exercise tolerance may benefit HF patients. Although studies on the DPP-4-GLP-1 pathway and exercise tolerance are limited, previous studies indicated that GLP-1 can recruit microvasculature to skeletal muscle [9] and induce mitochondrial activity, which enhances O_2_ consumption [10, 11]. Additionally, a study indicated that a DPP-4 inhibitor improved exercise tolerance and mitochondrial biogenesis by activating GLP-1 receptor signalling [12]. The greater peak O_2_ consumption is related to better exercise tolerance [13].

Dipeptidyl peptidase-4 (DPP-4) inhibitors and glucagon-like peptide-1 receptor agonists (GLP-1 RAs) are used to treat type 2 diabetes mellitus (T2DM) in adults [14, 15]. Glucagon-like peptide-1 (GLP-1) is an incretin hormone released from the gut, and it can be cleaved by dipeptidyl peptidase-4 within 2 min [16, 17]. Inhibiting DDP-4 can prolong the action of GLP-1, while a GLP-1 RA is a peptide similar to GLP-1 and can resist degradation by DPP-4. Thus, both DPP-4 inhibitors and GLP-1 RAs have the same pharmacological effect of prolonging the activation of the GLP-1 receptor [18]. A previous study indicated that abnormal glucose metabolism was associated with exercise intolerance [19], and recently, some clinical trials investigated the effect of these drugs in HF patients [4, 20–23]. It is worth investigating whether DPP-4 inhibitors/GLP-1 RAs improved exercise tolerance in HF patients.

As the effect of targeting the DPP-4-GLP-1 pathway on exercise tolerance in humans with HF remains unclear, we performed this systematic review and meta-analysis to investigate whether DPP-4 inhibitors/GLP-1 RAs could improve exercise tolerance in HF patients. Short-term treatment may have limited efficacy, as GLP-1 agonist treatment is usually initiated at low doses and increases to higher doses based on clinical response, with a likely dosage adjustment after 1 month [24, 25]; thus, a minimum treatment duration of 1 month will be needed.

## Methods

### Search strategy

We performed this systematic review and meta-analysis based on the Preferred Reporting Items for Systematic Reviews and Meta-Analyses (PRISMA) statement [26]. An electronic search in the PubMed, EMBASE and Cochrane Library databases was carried out through March 8th, 2019, for eligible trials with no language restrictions. The search strategy mixed medical subject heading (MeSH) and free-text words (*Dipeptidyl Peptidase IV Inhibitors OR Alogliptin OR Anagliptin OR Linagliptin OR Saxagliptin OR Sitagliptin OR Vildagliptin) OR (Glucagon-like peptide 1 receptor agonist OR Albiglutide OR Exenatide OR Dulaglutide OR Semaglutide OR Liraglutide OR Lixisenatide OR Taspoglutide OR Benaglutide) AND (Heart Failure) AND (RCT Filter*). The details are shown in Additional file 1. A manual search of references of systematic reviews and meta-analyses was also performed.

### Eligibility criteria

Randomized, controlled trials were included if they involved adults aged 18 years or over who had HF. The intervention group needed to treat patients with glucagon-like peptide-1 agonist or dipeptidyl peptidase-4 inhibitor, and the minimum duration of treatment needed to be no less than 1 month.

Trials were excluded if they (1) were ongoing trials; (2) did not have a full text; (3) were randomized crossover trials; and (4) did not measure exercise tolerance, including the 6-min walk test (6MWT) and/or peak O_2_ consumption.

### Study selection and data extraction

Two reviewers (Chen and Huang) selected records independently. Consultation with a third researcher was necessary to make a final determination when discrepancies occurred. Data were extracted from eligible trials with a data collection form that had been prepared previously. The following data were collected:

(1) Authors, years of publication, and study design; (2) Sample size, sex ratio, age, and the New York Heart Association (NYHA) functional class; (3) intervention setting and duration of treatment; (4) type 2 diabetes mellitus (T2DM) status, additional anti-diabetic drugs, and myocardial infarction history; (6) baseline and end-point characteristics of exercise tolerance assessment (6MWT, peak O_2_ consumption) and QoL; and (7) all-grade AEs and all-cause deaths. If more than one paper was published using data from the same trial, data from the longest follow-up associated with our primary outcomes or secondary outcomes were extracted.

All data were transformed to mean_change_ ± standard deviation (SD)_change_ when the data followed a normal distribution. The following formula was used:

$$ {\mathrm{SD}}_{\mathrm{change}}=\mathrm{square}\ \mathrm{root}\ \left[{\left({\mathrm{SD}}_{\mathrm{pre}-\mathrm{treatment}}\right)}^2+{\left({\mathrm{SD}}_{\mathrm{post}-\mathrm{treatment}}\right)}^2-\left(2\mathrm{R}\times {\mathrm{SD}}_{\mathrm{pre}-\mathrm{treatment}}\times {\mathrm{SD}}_{\mathrm{post}-\mathrm{treatment}}\right)\right] $$

R can be determined by the full data that were reported in a similar study, and it can be calculated by the formula [27].

$$ \mathrm{R}=\left[\left({\mathrm{SD}}_{\mathrm{pre}-\mathrm{treatment}}\right)2+{\left({\mathrm{SD}}_{\mathrm{post}-\mathrm{treatment}}\right)}^2-{\left({\mathrm{SD}}_{\mathrm{change}}\right)}^2\right]/\left[2\times {\mathrm{SD}}_{\mathrm{pre}-\mathrm{treatment}}\times {\mathrm{SD}}_{\mathrm{post}-\mathrm{treatment}}\right] $$

If the relevant data were unavailable, R could be assumed to be 0.5 [28].

If the trial reported only 95% CI without the SD, we used the formula below to infer the SD.

$$ 95\%\mathrm{CI}=\mathrm{mean}\pm 1.96\mathrm{SE} $$

SD = SE × square root (N), where N represents the sample size of the trial.

### Data analysis

We used RevMan 5.3 (The Nordic Cochrane Centre) to conduct the meta-analysis, and pooled mean_change_ and SD_change_ were obtained as summary data. If an outcome was measured by different methods, we used the standardized mean difference (SMD) or applied the mean difference (MD). The results were considered statistically significant when *P* values ≤0.05 were presented in forest plots. The I^2^ test was used to evaluate heterogeneity. Values ranged from 0% (homogeneity) to 100% (high heterogeneity), and I^2^ ≥ 50% indicated significant heterogeneity. If the heterogeneity was not significant, the results from the fixed-effects model and the random-effects model were similar. When the heterogeneity was significant, the random-effects model was used.

The primary outcome was exercise tolerance, which was measured in a standardized way by a 6-min walk test (6MWT) and peak O_2_ consumption [29, 30]. Secondary outcomes included QoL, AEs and all-cause death.

### Risk of bias and sensitivity analysis

We used the Cochrane Collaboration’s tool to assess the risk of bias by two reviewers independently (Chen and Huang). To test the stability of the results, a range of sensitivity analyses were carried out: 1) an analysis including only trials using the double-blinding method, as open-label trials have a high risk of performance bias; 2) an analysis using different R values [28, 30]; and 3) a meta-analysis of QoL including only the trials using the Minnesota Living with HF Questionnaire (MQOL), as it has the opposite interpretation as the Kansas City Cardiomyopathy Questionnaire (KCCQ).

## Results

**Fig. 1:**
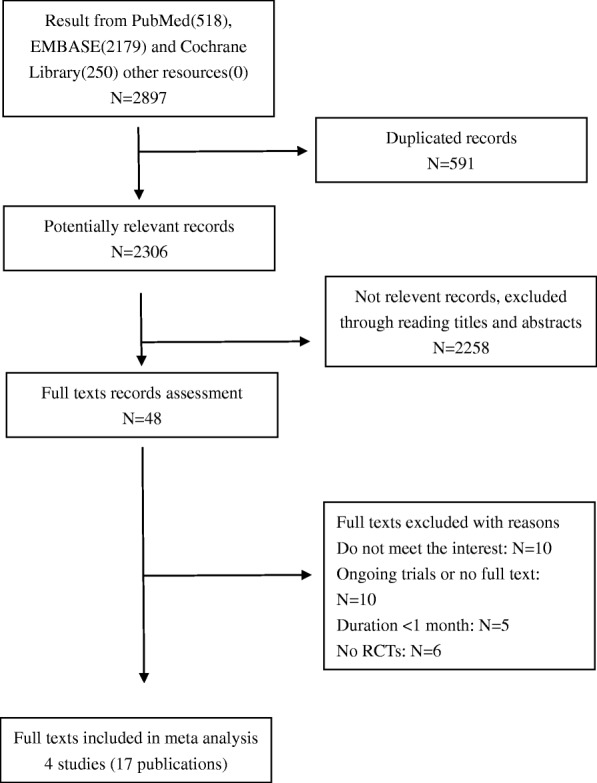
Process of selecting trials

A total of 2897records from eligible trials were identified and selected, as shown in Fig. 1. Five hundred ninety-one records were excluded because of duplication. After screening titles and abstracts, 2258 irrelevant records were excluded, and the full texts of the remaining 48 papers were assessed. Consequently, 4 trials (17 publications) met the eligibility criteria in the end.

### Study characteristics

The characteristics of the 4 included trials are shown in Table 1. These trials were all published in 2016, and the sample sizes were between 36 and 300, with the duration of treatment ranging from 12 weeks to 52 weeks. One trial followed an open-label design, and the remaining 3 were double-blinded trials. Of all four eligible trials, 1 trial investigated both GLP-1 agonists and DPP-4 inhibitors [22], and 3 trials studied GLP-1 agonists [4, 20, 21]. In particular, 1 trial included only patients with a previous history of acute myocardial infarction [22]. Four studies reported pre-intervention and post-intervention or 6MWT_change_ and QoL_change_, and 1 trial reported peak O_2_ consumption.

**Table 1:** Study characteristics

Author, years	Study design	Patients		Intervention
Arturi2016	Open-label RCT	Total 32 participants TG_liraglutide_: *N* = 10 (70% male) 59.5 ± 9 years, LVEF 41.5 ± 2.2% TG_sitagliptin:_*N* = 10 (60% male) 60.5 ± 10 years, LVEF 41.8 ± 2.6% CG: *N* = 12 (75% male) 60 ± 8 years LVEF 42 ± 1.5%, Of all patients, 27 with NYHA class III and 5 with NYHA class II		liraglutide 1.8 mg VS sitagliptin 100 mg VS glargine insulin
Jorsal2016	Double-blinded RCT	Total 241 participates TG: *N* = 122 (89%male) 65 ± 9.2 years LVEF 33.7 ± 7.6% NYHA class I-III CG: *N* = 119(89%male) 65 ± 10.7 years LVEF35.4 ± 9.4% NYHA class I-III		liraglutide 1.8 mg VS placebo
Lepore2016	Double-blinded RCT	Total 82 participates TG:*N* = 27 (74%male) 58 ± 10 years LVEF 31 ± 8.3% NYHA class II-III CG: *N* = 30 (70%male) 56 ± 10 years LVEF 32 ± 8.6% NYHA class II-III 25 participates in 3.75 mg or 15 mg was discontinued		Albiglutide 3.75 mg,15 mg,30 mg VS placebo
Margulies2016	Double-blinded RCT	Total 300 participates TG:*N* = 154(80%male) 62 (52–68) years LVEF 25(20–33)% NYHA class II-IV CG:*N* = 146 (77%male) 61 (51–67) years LVEF 25 (19–32)% NYHA class II-IV		liraglutide; at 0.6 mg SQ daily for 7 days, 1.2 mg SQ daily from day 7 through day 30, 1.8 mg for the rest VS placebo
Author, years	Follow-up	T2DM status	Additional antidiabetic drugs	Myocardial infraction history
Arturi2016	52 weeks	T2DM	Metformin or metformin and sulfonylurea	Prior acute myocardial infarction
Jorsal2016	24 weeks	With or without T2DM	Any anti-diabetes drugs except GLP-1 receptor agonists, glitziness, pramlintide or any DPP-4 inhibitor within 30 days prior to randomization. Patients treat with fast-acting insulin were also excluded	Myocardial infarction within 3 months were excluded
Lepore2016	12 weeks	Non-T2DM	NR	No myocardial infarction
Margulies2016	180 days	With or without T2DM	Ongoing GLP-1 agonists or DDP-4 inhibitors or thiazolidinedione are not allowed. If patients treated with DDP-4, washed out for a week was permitted	NR

*NYHA class* New York Heart Association functional class, *T2DM* Type 2 diabetes mellitus, *TG* Test group, *CG* Control group, *NR* Not reported

### Risk of bias assessment

**Fig. 2:**
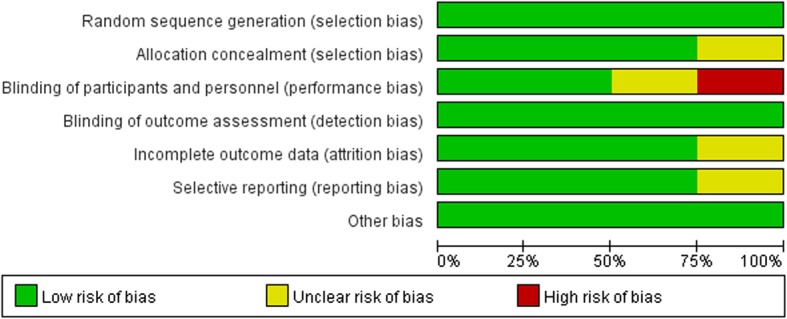
Risk of bias graph of each trial

**Fig. 3:**
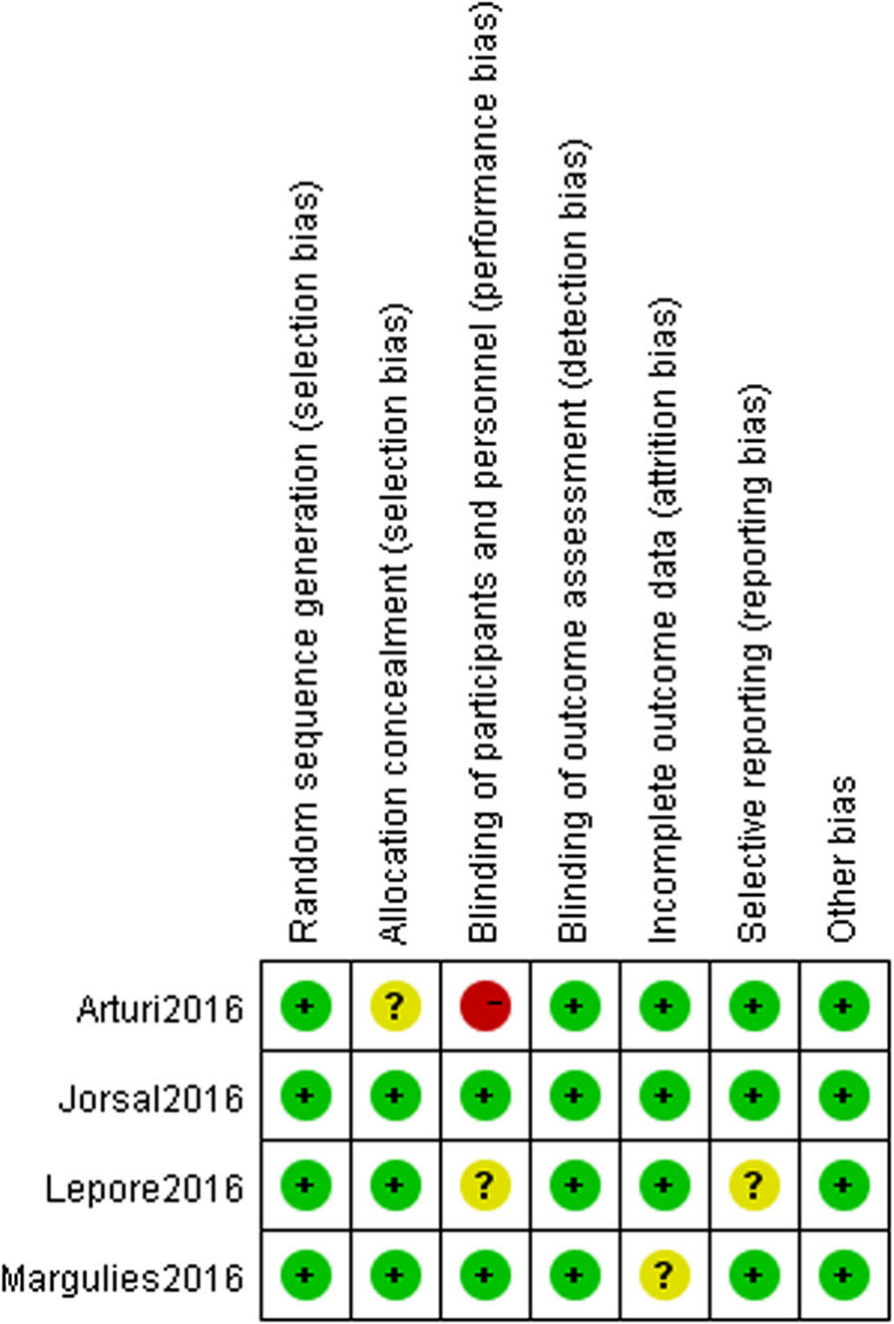
Risk of bias summary of each trial

The risk of bias is shown in Figs. 2 and 3. In an open-label trial [22], echocardiographs were read in a random order by an investigator who had no knowledge of the patients’ blood pressure or other clinical data, so we determined blinding of outcome assessment as low risk. The reasons for each evaluation are shown in Additional file 2.

## Outcomes

Of the 4 trials, outcomes were presented as the mean (95% CI) in one trial [21] and as the mean ± SD/SE in 3 trials [4, 20, 22]. All data were transformed into the mean_change_ ± SD_change_. R was determined according to Lepore (2016) [20], as it reports pre-intervention outcomes, post-intervention outcomes and changes from baseline to the end-point. We also performed a sensitivity analysis between different values of R (shown in Additional file 3). The results showed that R did not impact the results of the meta-analysis except for the lower heterogeneity when R = 0.5, so the following figures are displayed assuming R = 0.5. As the open-label trial may have a high risk of performance bias, we performed a sensitivity analysis by removing the Arturi (2016) study to confirm the stability of the results (shown in Additional file 3), and the analysis yielded similar results. Because AEs were reported with different methods and only 1 patient died among the 3 trials, we presented AE data in a summarized table and did not pool the data into a meta-analysis.

### 6 min walk test

**Fig. 4:**
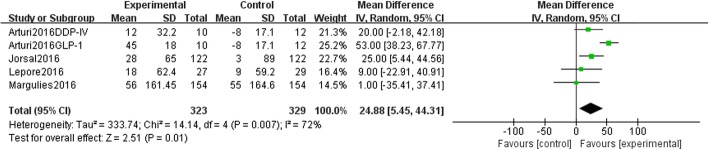
DPP-4 inhibitors/GLP-1 agonists VS insulin glargine/placebo in the 6MWT

The impact on the 6MWT was investigated in 4 trials [4, 20–22]. A total of 323 patients were randomly assigned to the experimental group, and 316 patients were assigned to the control group. In each trial, investigators calculated the walking distance in metres, so we used the MD in the meta-analysis. The results showed that there was a statistically significant difference between the two arms as shown in Fig. 4 [MD 24.88 (95% CI 5.45, 44.31), *P* = 0.01]. The sensitivity analysis confirmed the result (shown in Additional file 3); thus, DPP-4 inhibitors/GLP-1 RAs may benefit patients.

### Peak oxygen consumption

One trial [20] (including 27 patients in the experimental group and 29 patients in the control group) investigated peak O_2_ consumption. The results showed that peak O_2_ consumption in the experimental group improved significantly compared with that of the placebo group [MD 1.5(SE = 0.7), *P* = 0.024].

### Quality of life

**Fig. 5:**
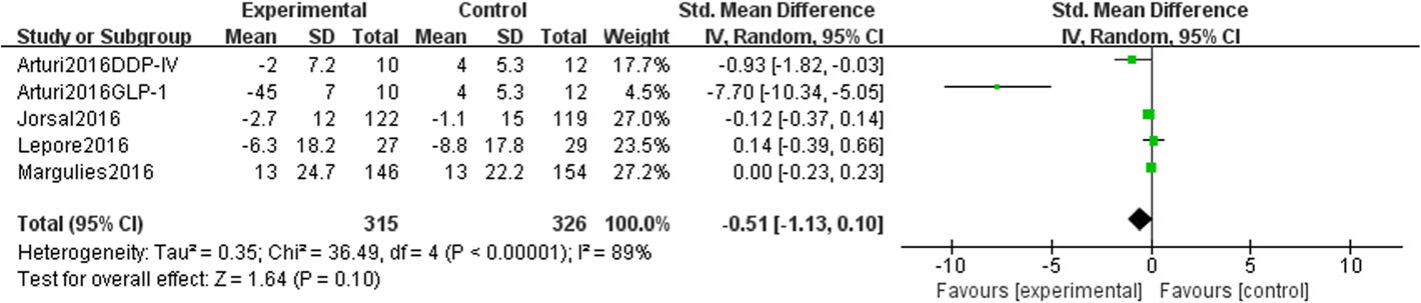
DPP-4 inhibitors/GLP-1 agonists VS insulin glargine/placebo in QoL

All eligible trials [4, 20–22] measured QoL. Three of the four used the MQOL [4, 20, 22], and one trial used the KCCQ [21]. A high score on the MQOL indicates low QoL, but a high score indicates high quality on the KCCQ. To pool these 4 trials into a meta-analysis, we put experimental data from Margulies (2016) into a “control group” and put control data into an “experimental group” in the forest plot. Because of the different methods, we applied SMD in this situation. The results showed no difference between the two groups as shown in Fig. 5 [SMD -0.51 (95% CI -1.13, 0.10), *P* = 0.10]. The sensitivity analysis showed that the treatment may improve patients’ QoL but not in a stable way (shown in Additional file 3). Therefore, it was determined that using DPP-4 inhibitors or GLP-1 agonists in HF patients did not affect QoL.

### AEs and all-cause death

The data were collected and are displayed in Table 2. Different methods were used to collect AE data in each trial; thus, we did not perform a meta-analysis. Of all 4 trials, one reported neither death nor AEs (mild nausea in liraglutide-treated patients could be solved without treatment) [22], and only one study demonstrated that the number of non-serious gastrointestinal events and central nervous system (CNS) events in the GLP-1 group were significantly higher than that in the placebo group; however, there was no difference in the incidence of serious adverse events (SAEs) [4]. Nonetheless, the rest of the studies did not report statistically significant AEs associated with GLP-1 RAs. Further studies are warranted to confirm the safety of GLP-1 RAs among HF patients.

**Table 2:** All-cause death and adverse events

	Treatment and control	Patients included in analysis	All cause death (N,)	Drug related adverse events (N)
Arturi2016	Liraglutide	10	0	0
	Sitagliptin	10	0	0
	Insulin glargine	12	0	0
Jorsal2016	Liraglutide	122	1	AE (cardiac = 13, gastrointestinal = 80*, CNS = 38*, other = 38(31.1%)), SAE (cardiac = 12, gastrointestinal = 0, CNS = 2, other = 13)
	Placebo	119	0	AE (cardiac = 10, gastrointestinal = 19, CNS = 15, other = 38), SAE (cardiac = 3, gastrointestinal = 1, CNS = 3, other = 9)
Lepore2016	Albiglutide	27	0	AE = 20, SAE = 0
	Placebo	30	0	AE = 25, SAE = 4
Margulies2016	Liraglutide	154	19	Cardiac = 37; glycemic = 36; other = 35
	Placebo	146	16	Cardiac = 34; glycemic = 28; other = 52

*means *P* ≤ 0.05 compared with placebo

The number of deaths in the control groups was 0 in 3 trials [4, 20, 22], and one patient died in the experimental group in 1 of the 3 trials [4]. Thus, we did not perform a meta-analysis. Another trial reported that 19 and 16 patients died in the experimental group and the control group, respectively, with no significant difference [21].

Overall, although we could assess the safety only through a table and not with statistical methods, DPP-4 inhibitors/GLP-1 agonists did not appear to increase mortality or SAE among HF patients.

## Discussion

Dyspnoea and fatigue, which are the most common symptoms among HF patients, can result in exercise intolerance [5]. Exercise intolerance can decrease health-related QoL and contribute to poor prognosis. The 6MWT is a common method used to measure patients’ exercise tolerance, while peak O_2_ consumption is another standard method [29, 30]. In this systematic review and meta-analysis, the results of the 6MWT and peak O_2_ consumption suggested that DPP-4 inhibitors/GLP-1 RAs improve patients’ exercise tolerance and did not reduce patients’ QoL, with high heterogeneity among the results. In accordance with the Cochrane Handbook, we conducted a sensitivity analysis using the leave-one-out method by removing trials with specific characteristics that may have caused unstable results. The study characteristics table clearly showed that the Arturi (2016) study was most likely to cause instability because it was an open-label trial, while the others were all double-blinded trials. Moreover, the Arturi (2016) study enrolled only patients with T2DM with a history of acute myocardial infarction, which is significantly different from the populations in the other trials. The sensitivity analysis confirmed the stability of our outcome, as shown in Additional file 1. Additionally, Margulies (2016) used the KCCQ to measure QoL, while the other studies used the MQOL. As these two methods have opposite interpretations, we pooled them into a meta-analysis that may have resulted in unstable results. Thus, a sensitivity analysis was also conducted, and the results confirmed that the treatment did not decrease patients’ QoL.

Although studies on the DPP-4-GLP-1 pathway and exercise tolerance are still limited, previous studies have demonstrated that GLP-1 can recruit microvasculature and induce mitochondrial activity in muscle [9, 10, 12]. Microvasculature is responsible for delivering oxygen, nutrients, and hormones to human skeletal muscle and removing metabolic products [31]. During exercise, the microvasculature plays a very important role in ensuring a sufficient supply of oxygen and nutrients; thus, the mitochondria can use oxygen and nutrients to generate ATP [13]. Targeting the DPP-4-GLP-1 pathway increases GLP-1 levels, thus recruiting microvasculature to muscle to ensure the supply of oxygen and nutrients and inducing mitochondrial activity. Better peak O_2_ consumption is correlated with better microvasculature function. Consequently, this series of reactions may result in improved exercise tolerance. Moreover, a study indicated that a DPP-4 inhibitor improved exercise tolerance by activating GLP-1 receptor signalling [12]. One trial reported peak O_2_ consumption in this systematic review, and the results showed that the experimental group was superior to the control group [20]. However, the authors of the trial indicated that it might be a spurious finding, as the 6MWT and QoL did not exhibit a significant improvement in this trial. In our meta-analysis, it was shown that the 6MWT could be improved after treatment. The 6MWT is another standard method to measure exercise tolerance in HF patients, and the result of the meta-analysis is more reliable than that of a single trial. Thus, we suggest that targeting the DPP-4-GLP-1 pathway benefits patients with limited exercise tolerance.

Our results did not show a significant improvement in health-related QoL after treatment. It is well recognized that QoL differs between sexes, ethnicities, age groups and so on [5]. Although improved exercise tolerance may affect QoL, the patient’s living environment or other health problems such as obesity do as well [32, 33]. It should be noted that GLP-1 RAs can be used only through intramuscular injection, and the inconvenience and pain of injection may contribute to this finding.

Although DPP-4 inhibitors/GLP-1 RAs have been widely used in T2DM patients worldwide, there is a concern about AEs, as the Savor-TIMI 53 trial reported that saxagliptin increased the rate of hospitalization for HF [34]. However, the later studies all demonstrated that DPP-4 inhibitors can reduce mortality and do not increase the incidence of AEs [35–37], and the same findings were observed for GLP-1 RAs [38, 39]. Our results also showed that the treatment did not appear to increase the incidence of all-cause death or SAEs. The reason the rate of hospitalization for HF increased in the Savor-TIMI 53 trial may be due to the enrolled patients’ basic disease, such as previous chronic kidney disease [40]. It should be noted that patients with a history of myocardial infarction (MI) may benefit from treatment. A trial in this systematic review, Arturi (2016), enrolled patients who had previous MI, and the results showed that patients can receive significant benefit from GLP-1 RA treatment. Previous studies have also indicated that this kind of patient may benefit from DPP-4 inhibitor treatment [41]. Chen, W. R. et al. demonstrated that liraglutide can improve left ventricular ejection fraction (LVEF) in patients with non-ST-segment-elevation myocardial infarction [42] and improve myocardial salvage and infarct size after ST-segment-elevation myocardial infarction [43]. Further studies on DPP-4 inhibitors and GLP-1 RAs in MI patients are needed.

Two trials did not report significant improvement, which may have been due to the patients’ characteristics. A recent study showed that serum DPP-4 may be associated with the effect of DPP-4 inhibitors, and patients with serum DPP-4 between 455.6 and 625.5 ng/mL may be the most likely to obtain treatment benefits, such as a 3-year reduction in all-cause mortality [44]. Unfortunately, these trials did not measure serum DPP-4 or GLP-1 levels, which is likely a predictor of treatment efficacy.

In this systematic review, we included trials investigating the effect of DPP-4 inhibitors or GLP-1 RAs on HF patients with or without T2DM. The results suggested that these inhibitors had a positive effect on exercise tolerance and did not decrease QoL or show signs of increasing the incidence of all-cause death or SAEs. Previous studies have demonstrated that DPP-4 inhibitors and GLP-1 RAs may be associated with cardiovascular benefits [45, 46]. DDP-4 inhibitors were reported to prevent cardiac diastolic dysfunction [47], ameliorate cardiac function in pressure overload heart failure [48], and attenuate the annual exacerbation of diastolic dysfunction [49]. Multiple lines of evidence have demonstrated the beneficial effects of GLP-1 RAs on heart function [50–52]. Meta-analyses have also suggested that these drugs can improve LVEF in patients with or without HF [53, 54]. Thus, targeting the DPP-4-GLP-1 pathway may be a potential strategy to improve heart function.

Exercise intolerance is well recognized in patients with T2DM and is known as a predictor of adverse prognosis [55]. Our study suggested that the use of DPP-4 inhibitors/GLP-1 RAs in HF patients with or without T2DM improves peak O_2_ consumption and 6MWT, contributing to better exercise tolerance. As the need for medical care in patients with HF and diabetes increases and the standard treatments are lacking [6], DPP-4 inhibitors and GLP-1 RAs, both anti-diabetic drugs, have shown beneficial effects in improving exercise tolerance in HF patients. Although these drugs have no indication for patients with HF, they may be better choices for patients with diabetes and HF. Furthermore, because this pathway contributes to improvement in exercise tolerance, it may be worth further investigation with exercise-intolerant patients with other diseases.

To our knowledge, this is the first systematic review and meta-analysis of randomized controlled trials to investigate the long-term treatment effect of DPP-4 inhibitors and GLP-1 RAs in HF patients. There are several limitations to this study. First, each trial had different collection methods for AEs. We could not perform a meta-analysis, but none of the studies reported an increased incidence of mortality or SAEs. Second, although DPP-4 inhibitors and GLP-1 RAs have the same pharmacological effect, DPP-4 inhibitors can be taken orally, which is more convenient than GLP-1 RAs, but we cannot compare the two types of drugs due to the limited number of trials. We hope that more studies can demonstrate the effect of DPP-4 inhibitors due to the convenience of oral drugs. Third, there were only 4 trials included in this meta-analysis, but these trials were all defined as high quality after assessment by the Cochrane Collaboration’s tool. The results of the sensitivity analyses also confirmed the stability of our results. Finally, although targeting DPP-4-GLP-1 signalling may improve exercise tolerance in HF patients, it has not been approved by any organizations, and we cannot draw a conclusion regarding whether type 2 diabetes mellitus patients with HF could benefit more from treatment. Thus, further study is necessary.

## Conclusion

DDP-4 inhibitors/GLP-1 RAs can improve exercise tolerance in HF patients, do not appear to increase the incidence of all-cause death or SAEs and do not decrease health-related QoL. As a result, current evidence shows that it may be a good choice for T2DM patients with HF.

## Supplementary information

**Additional file 1.** Search strategy, The search strategy in PubMed, EMBASE and Cochrane Library.

**Additional file 2.** Details of risk and bias assessment, This file included the supporting evidences of how we assessed the risk of bias.

**Additional file 3.** Sensitive analysis, We have conducted sensitive analyses of different study designed, R value and questionnaires, this file presented the results.

## Data Availability

The datasets used and/or analysed during the current study available from the corresponding author on reasonable request.

## Abbreviations

6MWT: 6-min walk test
AE: Adverse event
CNS: Central nervous system
DPP-4 inhibitor: Dipeptidyl peptidase-4 inhibitor
GLP-1 RA: Glucagon-like peptide 1 receptor agonist
HF: Heart failure
KCCQ: Kansas City cardiomyopathy questionnaire
MD: Mean difference
MI: Myocardial infarction
MQOL: Minnesota living with heart failure questionnaire
NYHA: New York heart association
QoL: Quality of life
SAE: Serious adverse event
SD: Standard deviation
SMD: Standardized mean difference
T2DM: Type 2 diabetes mellitus
