# Supplementary material for: Targeting the DPP-4-GLP-1 pathway improves exercise tolerance in heart failure patients: a systematic review and meta-analysis
Source: BMC Cardiovasc Disord. 2019 Dec 23;19:311. doi: 10.1186/s12872-019-01275-5 (PMC6927173; doi:10.1186/s12872-019-01275-5)
Supplement: Supplementary file 1 — Additional file 1. Search strategy, The search strategy in PubMed, EMBASE and Cochrane Library. [file 12872_2019_1275_MOESM1_ESM.pdf]

Supplementary 1, search strategy

Search strategy in PubMed

"Dipeptidyl-Peptidase IV Inhibitors"[Mesh]) OR Dipeptidyl-Peptidase IV Inhibitors OR Dipeptidyl Peptidase IV Inhibitors OR DPP 4 inhibitors OR DPP-IV inhibitors OR DPP-4 inhibitors OR DPP IV inhibitors OR Dipeptidyl Peptidase 4 Inhibitors OR Dipeptidyl-Peptidase 4 Inhibitors

OR

"alogliptin"[Supplementary Concept] OR oseni OR kazano OR nesina OR vipidia OR vipdomet OR Alogliptin

"anagliptin"[Supplementary Concept]) OR metoana OR anagliptin

"LC15-0444"[Supplementary Concept] OR gemigliptin

"Linagliptin"[Mesh] OR glyxambi OR jentadueto OR tradjenta

"2-(2,5-difluorophenyl)-5-(2-(methylsulfonyl)-2,6-dihydropyrrolo(3,4-c)pyrazol-5(4H)-yl)tetrahydro-2H-pyran-3-amine"[Supplementary Concept] OR omarigliptin

"saxagliptin"[Supplementary Concept]) OR kombiglyze OR onglyza OR qtern

"Sitagliptin Phosphate"[Mesh] OR Janumet OR Januvia OR juvisync OR steglujan OR janacti OR tesavel OR xelevia OR efficib OR velmetia OR ristfor OR ristaben

(("3-(4-(4-(3-methyl-1-phenyl-1H-pyrazol-5-yl)piperazin-1-yl)pyrrolidin-2 ylcarbonyl)thiazolidine"[Supplementary Concept]) OR teneligliptin))

"Vildagliptin"[Mesh] OR galvus OR jalra OR xiliarx OR eucreas OR icandra OR zomarist OR Vildagliptin

OR "Glucagon-Like Peptide 1"[Mesh] OR glucagon like peptide 1 receptor agonist OR glucagon-like peptide 1 receptor agonist OR glucagon-like peptide 1 agonist OR glucagon like peptide 1 agonist OR GLP-1 agonist OR GLP-1 receptor agonist OR GLP-1 Ra

OR

"rGLP-1 protein"[Supplementary Concept] OR Albiglutide OR tanzeum OR eperzan

"Exenatide"[Mesh] OR budureon OR byetta OR Exenatide

"dulaglutide"[Supplementary Concept] OR Trulicity OR Dulaglutide

"semaglutide"[Supplementary Concept] OR ozempic OR Semaglutide

"Liraglutide"[Mesh] OR Victoza OR saxenda OR xultophy OR levemir OR Liraglutide

"lixisenatide"[Supplementary Concept] OR adlyxin OR soliqua OR lyxumia OR suliqua OR Lixisenatide

"taspoglutide"[Supplementary Concept] OR taspoglutide

benaglutide

AND "Heart Failure"[Mesh] OR heart failure OR cardiac failure OR heart decompensation OR myocardial failure OR HF

AND "randomized controlled trial"[pt] OR "controlled clinical trial"[pt] OR randomized[tiab] OR placebo[tiab] OR "drug therapy"[sh] OR randomly[tiab] OR trial[tiab] OR groups[tiab]

RCT filter from: https://guides.library.harvard.edu/c.php?g=309982&p=2079544

Search strategy in EMBASE

**#1**	**'taspoglutide'/exp OR 'taspoglutide'**
**#2**	'exendin 4'/exp OR 'budureon' OR 'byetta' OR 'exenatide'
**#3**	'lixisenatide'/exp OR 'lixisenatide' OR 'adlyxin' OR 'soliqua' OR 'lyxumia' OR 'suliqua'
**#4**	'albiglutide'/exp OR 'albiglutide' OR 'tanzeum' OR 'eperzan'
**#5**	'liraglutide'/exp OR 'liraglutide' OR 'victoza' OR 'saxenda' OR 'xultophy' OR 'levemir'
**#6**	'dulaglutide'/exp OR 'dulaglutide' OR 'trulicity'
**#7**	'semaglutide'/exp OR 'semaglutide' OR 'ozempic'
**#8**	benaglutide
**#9**	#1 OR #2 OR #3 OR #4 OR #5 OR #6 OR #7 OR #8
**#10**	'glucagon like peptide 1 receptor agonist'/exp OR 'glucagon like peptide 1 receptor agonist' OR 'glucagon-like peptide 1 receptor agonist' OR 'glucagon-like peptide 1 agonist' OR 'glucagon like peptide 1 agonist' OR 'glp-1 agonist' OR 'glp-1 receptor agonist' OR 'glp-1 ra'
**#11**	#9 OR #10
**#12**	'alogliptin'/exp OR 'alogliptin' OR 'oseni' OR 'kazano' OR 'nesina' OR 'vipidia' OR 'vipdomet'
**#13**	'anagliptin'/exp OR 'anagliptin' OR 'metoana'
**#14**	'linagliptin'/exp OR 'linagliptin' OR 'glyxambi' OR 'jentadueto' OR 'tradjenta'
**#15**	'saxagliptin'/exp OR 'saxagliptin' OR 'kombiglyze' OR 'onglyza' OR 'qtern'
**#16**	'sitagliptin'/exp OR'sitagliptin' OR 'janumet' OR 'januvia' OR 'juvisync' OR 'steglujan' OR 'janacti' OR 'tesavel' OR 'xelevia' OR 'efficib' OR 'velmetia'OR 'ristfor' OR 'ristaben'
**#17**	'vildagliptin'/exp OR 'vildagliptin' OR 'galvus' OR 'jalra' OR 'xiliarx' OR 'eucreas' OR 'icandra' OR 'zomarist'
**#18**	'gemigliptin'/exp OR 'gemigliptin' OR 'omarigliptin'/exp OR 'omarigliptin' OR 'teneligliptin'/exp OR 'teneligliptin'
**#19**	#12 OR #13 OR #14 OR #15 OR #16 OR #17 OR #18
**#20**	'dipeptidyl peptidase iv inhibitor'/exp OR 'dipeptidyl peptidase iv inhibitor' OR 'dipeptidyl-peptidase iv inhibitor' OR 'dipeptidyl-peptidase 4 inhibitor' OR 'dipeptidyl peptidase 4 inhibitor' OR 'dpp 4 inhibitor' OR 'dpp-4 inhibitor' OR 'dpp-iv inhibitor' OR 'dpp iv inhibitor'
**#21**	#19 OR #20
**#22**	#11 OR #21
**#23**	'heart failure'/exp OR 'heart failure' OR 'cardiac failure' OR 'heart decompensation' OR 'myocardial failure' OR 'hf'
**#24**	#22 AND #23
**#25**	'randomized controlled trial'/exp OR 'controlled clinical trial'/exp OR randomized:ti,ab OR placebo:ti,ab OR 'drug therapy':lnk OR randomly:ti,ab OR trial:ti,ab OR groups:ti,ab
**#26**	#24 AND #25

RCT filter from: https://guides.library.harvard.edu/c.php?g=309982&p=2079544

Search strategy in Cochrane Library

#1	MeSH descriptor: [Liraglutide] explode all trees
#2	(Liraglutide):ti,ab,kw OR (Victoza):ti,ab,kw OR (saxenda):ti,ab,kw OR (xultophy):ti,ab,kw OR (levemir):ti,ab,kw
#3	(Albiglutide):ti,ab,kw OR (tanzeum):ti,ab,kw OR (eperzan):ti,ab,kw
#4	(Exenatide):ti,ab,kw OR (budureon):ti,ab,kw OR (byetta):ti,ab,kw
#5	(dulaglutide):ti,ab,kw OR (Trulicity):ti,ab,kw
#6	(semaglutide):ti,ab,kw OR (ozempic):ti,ab,kw
#7	(lixisenatide):ti,ab,kw OR (adlyxin):ti,ab,kw OR (soliqua):ti,ab,kw OR (lyxumia):ti,ab,kw OR (suliqua):ti,ab,kw
#8	(taspoglutide):ti,ab,kw OR (benaglutide):ti,ab,kw (Word variations have been searched)
#9	#1 OR #2 OR #3 OR #4 OR #5 OR #6 OR #7 OR #8
#10	(glucagon like peptide 1 receptor agonist):ti,ab,kw OR (glucagon-like peptide 1 receptor agonist):ti,ab,kw OR (glucagon-like peptide 1 agonist):ti,ab,kw OR (glucagon like peptide 1 agonist):ti,ab,kw OR (GLP-1 agonist):ti,ab,kw
#11	(GLP-1 receptor agonist):ti,ab,kw OR (GLP-1 Ra):ti,ab,kw
#12	#10 OR #11
#13	#9 OR #12
#14	MeSH descriptor: [Dipeptidyl-Peptidase IV Inhibitors] explode all trees
#15	(Dipeptidyl-Peptidase IV Inhibitors):ti,ab,kw OR (Dipeptidyl Peptidase IV Inhibitors):ti,ab,kw OR (DPP 4 inhibitors):ti,ab,kw OR (DPP-IV inhibitors):ti,ab,kw OR (DPP-4 inhibitors):ti,ab,kw
#16	(DPP IV inhibitors):ti,ab,kw OR (Dipeptidyl Peptidase 4 Inhibitors):ti,ab,kw OR (Dipeptidyl-Peptidase 4 Inhibitors):ti,ab,kw
#17	#14 OR #15 OR #16
#18	(alogliptin):ti,ab,kw OR (oseni):ti,ab,kw OR (kazano):ti,ab,kw OR (nesina):ti,ab,kw OR (vipidia):ti,ab,kw
#19	(vipdomet):ti,ab,kw
#20	(metoana):ti,ab,kw OR (anagliptin):ti,ab,kw
#21	MeSH descriptor: [Linagliptin] explode all trees
#22	(Linagliptin):ti,ab,kw OR (glyxambi):ti,ab,kw OR (jentadueto):ti,ab,kw OR (tradjenta):ti,ab,kw
#23	(saxagliptin):ti,ab,kw OR (kombiglyze):ti,ab,kw OR (qtern):ti,ab,kw OR (onglyza):ti,ab,kw
#24	MeSH descriptor: [Sitagliptin Phosphate] explode all trees
#25	(Sitagliptin):ti,ab,kw OR (Janumet):ti,ab,kw OR (Januvia):ti,ab,kw OR (juvisync):ti,ab,kw OR (janacti):ti,ab,kw
#26	(tesavel):ti,ab,kw OR (xelevia):ti,ab,kw OR (efficib):ti,ab,kw OR (velmetia):ti,ab,kw OR (ristfor):ti,ab,kw
#27	(ristaben):ti,ab,kw
#28	(Vildagliptin):ti,ab,kw OR (galvus):ti,ab,kw OR (jalra):ti,ab,kw OR (xiliarx):ti,ab,kw OR (eucreas):ti,ab,kw
#29	(icandra):ti,ab,kw OR (zomarist):ti,ab,kw OR (teneligliptin):ti,ab,kw

	OR (omarigliptin):ti,ab,kw OR (gemigliptin):ti,ab,kw
#30	#18 OR #19 OR #20 OR #21 OR #22 OR #23 OR #24 OR #25 OR #26 OR #27 OR #28 OR #29
#31	#17 OR #30
#32	MeSH descriptor: [Heart Failure] explode all trees
#33	(Heart Failure):ti,ab,kw OR (cardiac failure):ti,ab,kw OR (heart decompensation):ti,ab,kw OR (myocardial failure):ti,ab,kw OR (HF):ti,ab,kw
#34	#32 OR #33
#35	#13 OR #31
#36	#34 AND #35
