# Supplementary material for: Targeting the DPP-4-GLP-1 pathway improves exercise tolerance in heart failure patients: a systematic review and meta-analysis
Source: BMC Cardiovasc Disord. 2019 Dec 23;19:311. doi: 10.1186/s12872-019-01275-5 (PMC6927173; doi:10.1186/s12872-019-01275-5)
Supplement: Supplementary file 2 — Additional file 2. Details of risk and bias assessment, This file included the supporting evidences of how we assessed the risk of bias. [file 12872_2019_1275_MOESM2_ESM.pdf]

# Supplementary 2, details of risk of bias assessment

1. **Arturi, F., et al., *Liraglutide improves cardiac function in patients with type 2 diabetes and chronic heart failure.* Endocrine, 2016. 57(3): p. 464-473.**

| Bias | Authors' judgement | Support for judgement |
|---|---|---|
| **Random sequence generation (selection bias)** | Low risk | Quote form publication: "randomization were carried out with the Research Randomizer tool，available online at www.randomizer.org" |
| **Allocation concealment (selection bias)** | Unclear risk | Comment：method to conceal allocation sequence not described |
| **Blinding of participants and personnel (performance bias)** | High risk | This was a single-center, open-label, randomized, 52-week active-comparator, parallel-group, pilot study |
| **Blinding of outcome assessment (detection bias)** | Low risk | Echocardiographic readings were made in random order by the investigator, who had no knowledge of patients' blood pressure and other clinical data |
| **Incomplete outcome data (attrition bias)** | Low risk | None of the patients presented severe hypoglycemic events and had the need for hospitalization for worsening CHF during the treatment period" |
| **Selective reporting (reporting bias)** | Low risk | "Analysis of variance (ANOVA) with post hoc Bonferroni correction for multiple comparisons was used to compare differences of continuous variables between groups"<br><br>"none of the patients<br>experienced adverse events or side effects with the exception of mild nausea in liraglutide-treated patients, which resolved spontaneously after a few days without requiring dose adjustment. None of the patients presented severe hypoglycemic events and had the need for hospitalization for worsening CHF during the treatment period" |
| **Other bias** | Low risk | none detected |

2. **Jorsal, A., et al.,** ***Effect of liraglutide, a glucagon-like peptide-1 analogue, on left ventricular function in stable chronic heart failure patients with and without diabetes (LIVE)-a multicentre, double-blind, randomised, placebo-controlled trial.*** **LIVE Eur J Heart Fail, 2016. 19(1): p. 69-77.**

| Bias | Authors' judgement | Support for judgement |
|---|---|---|
| Random sequence generation (selection bias) | Low risk | in Jorsal 2014(protocol)<br><br>"Simple randomization will occur consecutively in both groups according to a computer-generated randomization list in a 1:1 randomization ratio" |
| Allocation concealment (selection bias) | Low risk | in Jorsal 2014(protocol)<br><br>"Treatment allocation is web based and can be unblinded in case of medical emergencies if deemed necessary by the investigator. Unblinding can be made individually so that treatment blinding of other patients in the group remains unaffected" |
| Blinding of participants and personnel (performance bias) | Low risk | The LIVE study is an investigator-initiated, Danish multicenter, randomized, double-blinded, parallel, placebo controlled intervention trial |
| Blinding of outcome assessment (detection bias) | Low risk | An unblinded safety monitoring committee comprising independent cardiologists and endocrinologists evaluated data on serious adverse events and the study patients' safety. |
| Incomplete outcome data (attrition bias) | Low risk | "The study was designed to randomize 240 patients with an expected 20% dropout rate"<br><br>Comment: in the end of trial, each group have 106 patients had completed data |
| Selective reporting (reporting bias) | Low risk | primary outcome is difference in LVEF from baseline to follow-up, secondary outcome included Systolic function measured, End-systolic volume, Grade of diastolic function,6MWT,NT-proBNP,Blood pressure, Quality of life, Hospitalization for CHF, Mortality(2014 protocol). hospitalization for CHF did not report in Jorsal2016, but this can not lead to bias after reviewer's consideration. |
| Other bias | Low risk | Comment: none detected |

3. Lepore, J.J., et al., *Effects of the Novel Long-Acting GLP-1 Agonist, Albiglutide, on Cardiac Function, Cardiac Metabolism, and Exercise Capacity in Patients With Chronic Heart Failure and Reduced Ejection Fraction.* **JACC Heart Fail, 2016. 4(7): p. 559-566.**

| Bias | Authors' judgement | Support for judgement |
|---|---|---|
| Random sequence generation (selection bias) | Low risk | This was a multicenter, randomized, parallel arm, placebo-controlled study<br><br>The study was conducted in accordance with “good clinical practice” and all applicable regulatory requirements |
| Allocation concealment (selection bias) | Low risk | Subjects, investigators, and sponsor staff at the study sites were blinded to treatment allocation |
| Blinding of participants and personnel (performance bias) | Unclear risk | "Subjects, investigators, and sponsor staff at the study sites were blinded to treatment allocation; sponsor internal staff and staff from the contract research organization (Pharmaceutical Product Development, Wilmington, North Carolina) were unblinded" |
| Blinding of outcome assessment (detection bias) | Low risk | "Subjects, investigators, and sponsor staff at the study sites were blinded to treatment allocation"<br><br>This was a multicenter, randomized, parallel arm, placebo-controlled study<br><br>The study was conducted in accordance with “good clinical practice” and all applicable regulatory requirements |
| Incomplete outcome data (attrition bias) | Low risk | Eighty-one of 82 randomized subjects (97%) completed the study. One placebo-treated subject withdrew before the follow-up visit. |
| Selective reporting (reporting bias) | Unclear risk | Comment: "Review of data from an early in-stream analysis suggested limited efficacy in subjects receiving albiglutide 3.75 or 15 mg weekly. Accordingly, per protocol, randomization into these arms was discontinued. But the end point characteristic of 3.75mg and 15mg groups did not report. |
| Other bias | Low risk | Comment: none detected |

**4. Margulies, K.B., et al., *Effects of Liraglutide on Clinical Stability Among Patients With Advanced Heart Failure and Reduced Ejection Fraction: A Randomized Clinical Trial.* Jama, 2016. 316(5): p. 500-8.**

| Bias | Authors' judgement | Support for judgement |
|---|---|---|
| Random sequence generation (selection bias) | Low risk | "Subjects will be randomized using procedures determined by the Coordinating Center (CC) to one of 2 treatment groups. A permuted block randomization method stratified by site will be used to ensure relatively equal distribution of subjects to each arm within each clinical site" |
| Allocation concealment (selection bias) | Low risk | Comment: "A permuted block randomization method stratified by site will be used to ensure relatively equal distribution of subjects to each arm within each clinical site, and it is a double-blinded trial. |
| Blinding of participants and personnel (performance bias) | Low risk | The FIGHT study is a randomized, double-blinded, placebo-controlled clinical trial<br><br>"Blinding is ensured by preparation of identically appearing placebo and active drug" |
| Blinding of outcome assessment (detection bias) | Low risk | Comment: "The investigative sites will be given access to the treatment code for their participants for emergency unblinding ONLY by calling the CC. In the rare event of necessary unblinding, the CC medical monitor must be contacted to discuss the case." |
| Incomplete outcome data (attrition bias) | Unclear risk | Comment:106 Continued liraglutide 44 Discontinued liraglutide (29 before reaching a clinical end point) 9 Adverse reaction,35 Other And 103 Continued placebo 42 Discontinued placebo (25 before reaching a clinical end point) 9 Adverse reaction 33 Other |
| Selective reporting (reporting bias) | Low risk | Comment:106 Continued liraglutide 44 Discontinued liraglutide (29 before reaching a clinical end point) 9 Adverse reaction 35 Other, but 154 Included in primary analysis. and 103 Continued placebo 42 Discontinued placebo (25 before reaching a clinical end point) 9 Adverse reaction 33 Other, but 146 Included in primary analysis |
| Other bias | Low risk | Comment: none detected |
