# Supplementary material for: Targeting the DPP-4-GLP-1 pathway improves exercise tolerance in heart failure patients: a systematic review and meta-analysis
Source: BMC Cardiovasc Disord. 2019 Dec 23;19:311. doi: 10.1186/s12872-019-01275-5 (PMC6927173; doi:10.1186/s12872-019-01275-5)

Supplementary 3, sensitive analysis

Sensitive analysis of different R (calculate R according to Lepore2016)

1. 6 minute-walk test, R=0.85

Study or Subgroup	Experimental Mean	Experimental SD	Experimental Total	Control Mean	Control SD	Control Total	Weight	Mean Difference IV, Random, 95% CI
arturi2016DDP-IV	12	19.5	10	-8	9.5	12	23.5%	20.00 [6.77, 33.23]
arturi2016GLP-1	45	10.7	10	-8	9.5	12	24.8%	53.00 [44.46, 61.54]
jorsal2016	28	65	122	3	89	122	21.1%	25.00 [5.44, 44.56]
lepore2016	18	62.4	27	9	59.2	29	16.1%	9.00 [-22.91, 40.91]
margulies2016	56	161.45	154	55	164.6	154	14.5%	1.00 [-35.41, 37.41]
**Total (95% CI)**			**323**			**329**	**100.0%**	**24.72 [3.85, 45.59]**

Heterogeneity: Tau² = 437.63; Chi² = 27.79, df = 4 (P < 0.0001); I² = 86%
Test for overall effect: Z = 2.32 (P = 0.02)

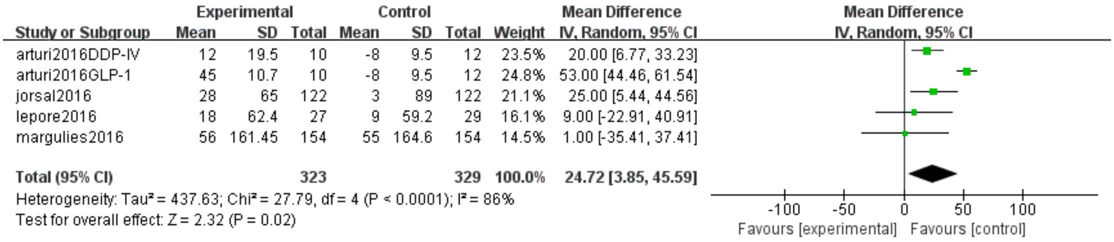

2. Quality of life, R=0.64

Study or Subgroup	Experimental Mean	Experimental SD	Experimental Total	Control Mean	Control SD	Control Total	Weight	Mean Difference IV, Random, 95% CI
arturi2016DDP-IV	-2	6.2	10	4	4.6	12	20.1%	-6.00 [-10.64, -1.36]
arturi2016GLP-1	-45	6.1	10	4	4.6	12	20.1%	-49.00 [-53.59, -44.41]
jorsal2016	-2.7	12	122	-1.1	15	119	20.2%	-1.60 [-5.03, 1.83]
lepore2016	3.5	18.2	27	-8.8	17.8	29	19.5%	12.30 [2.86, 21.74]
margulies2016	13	22.2	154	13	24.7	146	20.1%	0.00 [-5.32, 5.32]
**Total (95% CI)**			**323**			**318**	**100.0%**	**-9.00 [-29.08, 11.08]**

Heterogeneity: Tau² = 515.82; Chi² = 336.06, df = 4 (P < 0.00001); I² = 99%
Test for overall effect: Z = 0.88 (P = 0.38)

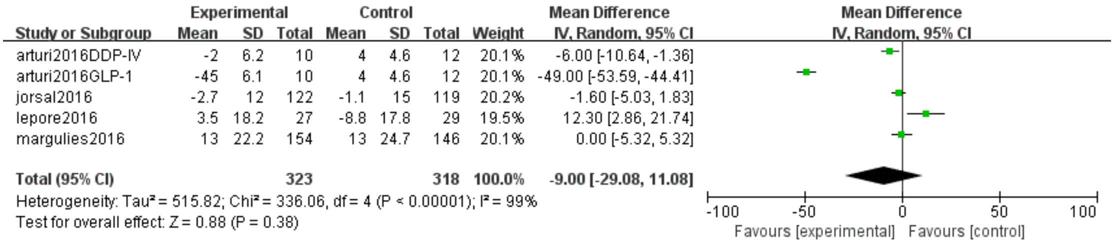

Sensitive analysis of removing Auturi2016(it was open-label trial and the rest of studies were double-blinded trials), assuming R=0.5

1. 6 minute-walk test

Study or Subgroup	Experimental Mean	Experimental SD	Experimental Total	Control Mean	Control SD	Control Total	Weight	Mean Difference IV, Random, 95% CI
arturi2016DDP-IV	12	32.2	10	-8	17.1	12	0.0%	20.00 [-2.18, 42.18]
arturi2016GLP-1	45	18	10	-8	17.1	12	0.0%	53.00 [38.23, 67.77]
jorsal2016	28	65	122	3	89	122	60.1%	25.00 [5.44, 44.56]
lepore2016	18	62.4	27	9	59.2	29	22.6%	9.00 [-22.91, 40.91]
margulies2016	56	161.45	154	55	164.6	154	17.3%	1.00 [-35.41, 37.41]
**Total (95% CI)**			**303**			**305**	**100.0%**	**17.23 [2.07, 32.39]**

Heterogeneity: Tau² = 0.00; Chi² = 1.63, df = 2 (P = 0.44); I² = 0%
Test for overall effect: Z = 2.23 (P = 0.03)

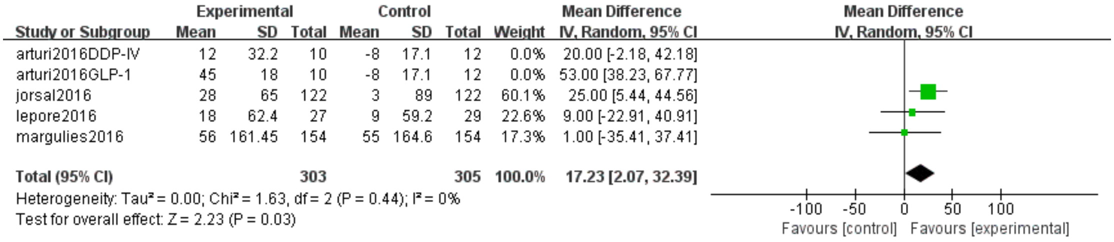

2. Quality of life

Study or Subgroup	Experimental Mean	Experimental SD	Experimental Total	Control Mean	Control SD	Control Total	Weight	Std. Mean Difference IV, Random, 95% CI
arturi2016DDP-IV	-2	7.2	10	4	5.3	12	0.0%	-0.93 [-1.82, -0.03]
arturi2016GLP-1	-45	7	10	4	5.3	12	0.0%	-7.70 [-10.34, -5.05]
jorsal2016	-2.7	12	122	-1.1	15	119	40.4%	-0.12 [-0.37, 0.14]
lepore2016	-6.3	18.2	27	-8.8	17.8	29	9.4%	0.14 [-0.39, 0.66]
margulies2016	13	24.7	146	13	22.2	154	50.3%	0.00 [-0.23, 0.23]
**Total (95% CI)**			**295**			**302**	**100.0%**	**-0.03 [-0.20, 0.13]**

Heterogeneity: Tau² = 0.00; Chi² = 0.91, df = 2 (P = 0.63); I² = 0%
Test for overall effect: Z = 0.42 (P = 0.67)

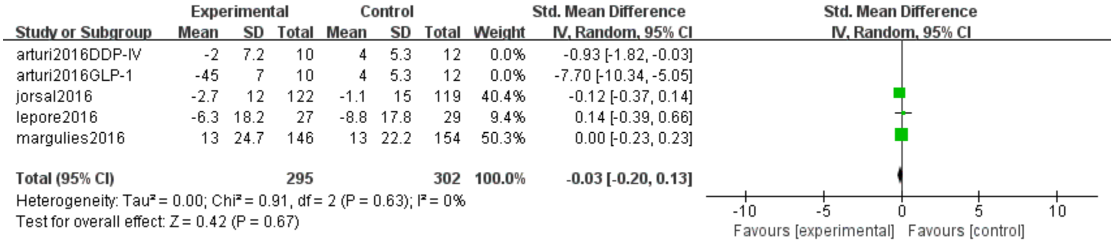

Sensitive analysis of removing Margulies2016(it used KCCQ and has the opposite interpretation of MQOL), assuming R=0.5

	Experimental			Control				Std. Mean Difference
Study or Subgroup	Mean	SD	Total	Mean	SD	Total	Weight	IV, Random, 95% CI
arturi2016DDP-IV	-2	7.2	10	4	5.3	12	26.8%	-0.93 [-1.82, -0.03]
arturi2016GLP-1	-45	7	10	4	5.3	12	11.6%	-7.70 [-10.34, -5.05]
jorsal2016	-2.7	12	122	-1.1	15	119	31.6%	-0.12 [-0.37, 0.14]
lepore2016	-6.3	18.2	27	-8.8	17.8	29	30.0%	0.14 [-0.39, 0.66]
margulies2016	13	24.7	146	13	22.2	154	0.0%	0.00 [-0.23, 0.23]
**Total (95% CI)**			**169**			**172**	**100.0%**	**-1.14 [-2.27, -0.01]**

Heterogeneity: Tau² = 1.04; Chi² = 35.31, df = 3 (P < 0.00001); I² = 92%
Test for overall effect: Z = 1.98 (P = 0.05)

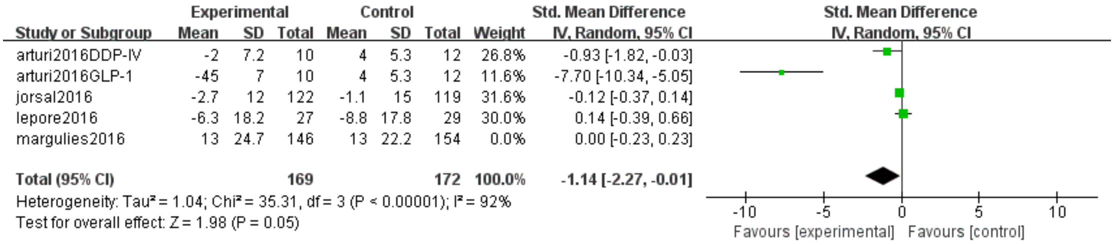

Sensitive analysis of removing both Margulies2016 and Auturi2016(different Questionnaire and blinding), assuming R=0.5

	Experimental			Control				Std. Mean Difference
Study or Subgroup	Mean	SD	Total	Mean	SD	Total	Weight	IV, Random, 95% CI
arturi2016DDP-IV	-2	7.2	10	4	5.3	12	0.0%	-0.93 [-1.82, -0.03]
arturi2016GLP-1	-45	7	10	4	5.3	12	0.0%	-7.70 [-10.34, -5.05]
jorsal2016	-2.7	12	122	-1.1	15	119	81.2%	-0.12 [-0.37, 0.14]
lepore2016	-6.3	18.2	27	-8.8	17.8	29	18.8%	0.14 [-0.39, 0.66]
margulies2016	13	24.7	146	13	22.2	154	0.0%	0.00 [-0.23, 0.23]
**Total (95% CI)**			**149**			**148**	**100.0%**	**-0.07 [-0.30, 0.16]**

Heterogeneity: Tau² = 0.00; Chi² = 0.73, df = 1 (P = 0.39); I² = 0%
Test for overall effect: Z = 0.60 (P = 0.55)

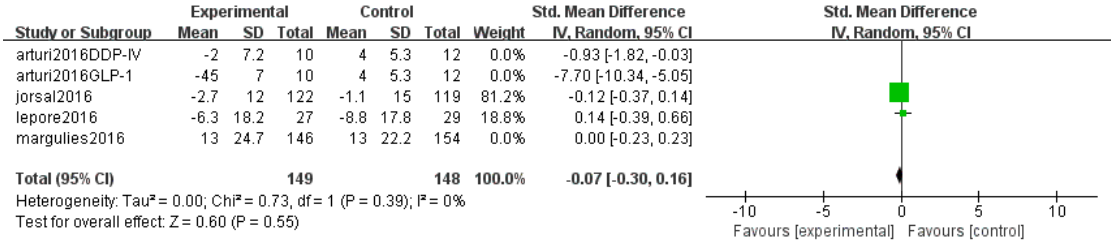

Supplement: Supplementary file 3 — Additional file 3. Sensitive analysis, We have conducted sensitive analyses of different study designed, R value and questionnaires, this file presented the results. [file 12872_2019_1275_MOESM3_ESM.pdf]
